# TEACHING ABOUT GERONTOLOGY AND AGING THROUGH INTERPROFESSIONAL EDUCATION AND COLLABORATION

**DOI:** 10.1093/geroni/igz038.1012

**Published:** 2019-11-08

**Authors:** Elaine T Jurkowski, Ruth Heitkamp, Cherie Kelly, Sharon Smaga

**Affiliations:** 1 Southern Illinois University Carbondale, Carbondale, Illinois, United States; 2 Center for Rural Health and Social Service Development, SIU School of Medicine, Carbondale, Illinois, United States; 3 Physician Assistant Program, Southern Illinois University Carbondale, Carbondale, Illinois, United States; 4 Family Practice Residency Program, SIU School of Medicine, Carbondale, Illinois, United States

## Abstract

Repeatedly as multidisciplinary professionals we are concerned about the individual patient we serve. Inter-professional collaboration across disciplines such as primary care medical practice, physician assistants, social workers and psychologists does not occur naturally, since educational programs are often taught independently of each other, but these disciplines are required to work collaboratively with each other. The objective was to promote communication across disciplines (Medicine, Social Work, Physician Assistant and Psychology) and help each discipline understand the roles played in promoting mental health and general health for older adults. An educational seminar was conducted using cases and guide questions focused on identifying strategies for care. The teams consisted of Medical Residents, a Social Work student, a Psychology student and a Physician Assistant student. A series of guide questions were provided, and teams were asked to discuss and identify a care plan. Debriefing followed to discuss the outcomes across all teams. Pre-post test results examined variables related to interdisciplinary collaboration. Findings suggest professionals were surprised at what they learned from the other disciplines they were collaborating with. They also learned about community based resources available as well as strategies to promote health outcomes. All participants felt that the opportunity to collaborate outside of their disciplines would strengthen their impact when working with older adults and their families. In conclusion, a problem based learning approach coupled with the opportunity to collaborate with other disciplines through (IPE) is a venue to improve overall collaboration across professionals and ultimately improve mental health outcomes of consumers.

